# Investigating Extracellular DNA Release in *Staphylococcus xylosus* Biofilm In Vitro

**DOI:** 10.3390/microorganisms9112192

**Published:** 2021-10-21

**Authors:** Sabine Leroy, Isabelle Lebert, Carine Andant, Pierre Micheau, Régine Talon

**Affiliations:** 1 Université Clermont Auvergne, INRAE, MEDIS, F-63000 Clermont-Ferrand, France; isabelle.lebert@inrae.fr (I.L.); carine.andant@inrae.fr (C.A.); pierre.micheau@inrae.fr (P.M.); regine.talon@inrae.fr (R.T.); 2 Université Clermont Auvergne, INRAE, VetAgro Sup, UMR EPIA, F-63122 Saint-Genès Champanelle, France; 3 Université Clermont Auvergne, INRAE, UNH, F-63000 Clermont-Ferrand, France

**Keywords:** *Staphylococcus xylosus*, biofilm, eDNA, cell lysis, amino sugar catabolism, DNA/RNA repair, protein turnover

## Abstract

*Staphylococcus xylosus* forms biofilm embedded in an extracellular polymeric matrix. As extracellular DNA (eDNA) resulting from cell lysis has been found in several staphylococcal biofilms, we investigated *S. xylosus* biofilm in vitro by a microscopic approach and identified the mechanisms involved in cell lysis by a transcriptomic approach. Confocal laser scanning microscopy (CLSM) analyses of the biofilms, together with DNA staining and DNase treatment, revealed that eDNA constituted an important component of the matrix. This eDNA resulted from cell lysis by two mechanisms, overexpression of phage-related genes and of *cidABC* encoding a holin protein that is an effector of murein hydrolase activity. This lysis might furnish nutrients for the remaining cells as highlighted by genes overexpressed in nucleotide salvage, in amino sugar catabolism and in inorganic ion transports. Several genes involved in DNA/RNA repair and genes encoding proteases and chaperones involved in protein turnover were up-regulated. Furthermore, *S. xylosus* perceived osmotic and oxidative stresses and responded by up-regulating genes involved in osmoprotectant synthesis and in detoxification. This study provides new insight into the physiology of *S. xylosus* in biofilm.

## 1. Introduction

*Staphylococcus xylosus* is a commensal species of the epithelium and mucous membranes of warm-blooded animals. It is frequently isolated from the skin of farm animals [1,2], hence its prevalence in foods of animal origin such as milk and milk products and fermented meat products [3,4]. Furthermore, *S. xylosus* colonizes the manufacturing environment of dry fermented sausage plants in relation to its ability to form biofilm [4,5]. Indeed, *S. xylosus* forms multilayered biofilm where cells are embedded in an extracellular polymeric matrix [5], which is a common trait in bacterial biofilms [6,7,8]. In *Staphylococcus epidermidis* and *Staphylococcus aureus*, one component of the matrix is designated as poly-N-acetylglucosamine polysaccharide (PNAG) whose production is governed by the *ica* operon [9,10]. In *S. xylosus*, however, exopolymer biosynthesis appears to be *ica*-independent and the composition of the polymer remains unknown [5,11]. Other surface components have been described as required for biofilm cohesion in *S. aureus* and *S. epidermidis*, namely, teichoic acids, the accumulation associated protein (Aap), and the biofilm-associated protein (Bap) [6,12,13,14,15]. In addition, the observation that certain strains of *S. epidermidis* and *S. aureus* form *ica*-independent biofilms has shown that extracellular DNA (eDNA) can serve as a natural glue connecting neighboring cells to each other [9,16,17,18]. In fact, eDNA is a component of the biofilm matrix of many bacterial species [19,20,21].

eDNA released from cells could result from autolysis. In *S. epidermidis* and *S. aureus*, eDNA in biofilm was released through the activity of the prominent murein hydrolase Atl [22,23]. In these two species, a two-component regulatory system LytSR affects murein hydrolase activity and autolysis [24]. LytSR regulates the expression of the *lrgAB* operon, which, together with the *cidABC* operon, has been shown to be a regulator in the control of cell death and lysis during biofilm development [25,26]. The *cidA* gene encodes a putative holin protein that is an effector of murein hydrolase activity and cell lysis [27], while *lrgA* encodes a putative antiholin that is an inhibitor of these processes [25]. Furthermore, in *S. aureus* CidR enhances *cidABC*, *lrgAB*, and *alsSD* (encoding proteins involved in acetoin production) expression in response to carbohydrate metabolism [28].

Other known mechanisms of eDNA release include phage-mediated cell death. Phage release has been observed in biofilms of both Gram-negative and Gram-positive bacteria [29,30,31]. In *Pseudomonas aeruginosa*, the phage Pf4 mediates the formation of small-colony variants in biofilms [29]. Lysogenic *S. aureus* cells in planktonic and biofilm cultures release phages into their surroundings; two morphologically distinct phages are observed [31]. The resulting lysis of cells in biofilm might promote the persistence of the remaining cells by furnishing nutrients.

In *S. xylosus*, genes encoding the holin/antiholin system and genes encoding phage proteins are highly overexpressed during growth in a meat model [32]. Moreover, a phage capsid protein is overexpressed in *S. xylosus* cultivated in biofilm compared to planktonic culture [11]. These results highlight that cell lysis could result in the release of DNA. Thus, the aim of this study was first to check if eDNA is released during biofilm formation by a microscopic approach and then to identify the mechanisms involved in cell lysis by a transcriptomic approach.

## 2. Materials and Methods

### 2.1. Bacterial Strain and Biofilm Formation

*S. xylosus* strain C2a expressing cyan fluorescent (C2a-B2) was used [33]. This strain contains the erythromycin-resistance plasmid pJEBAN2. The strain C2a-B2 was pre-cultivated in Brain Heart Infusion (BHI, Becton, Dickinson and Company, Le Pont de Claix, France) with 10 µg/mL erythromycin at 30 °C, with stirring at 170 rpm for 24 h. Bacterial concentration was then measured by determining the optical density at 600 nm (*OD*_600_) and appropriate dilution was prepared. The strain was then inoculated at 10^6^ CFU/mL in BHI in the Lab-Tek chamber slide system (1 chamber borosilicate cover glass system, NUNC 15536, 8.6 cm^2^) and incubated at 30 °C for 9, 24 and 48 h in a humid chamber. After incubation, the supernatant in the Lab-Tek chambers was removed, the adhered cells were washed twice with tryptone salt and then were detached by scratching in tryptone salt. The bacterial population of biofilm after 9, 24 and 48 h were determined by 10-fold serial dilutions on BHI agar plates and enumerated after 24-h culture at 30 °C. In parallel, the cells detached by scratching in tryptone salt were pelleted by centrifugation for 2 min at 4500× *g* and at 4 °C and the pellet was immediately frozen in liquid nitrogen and stored at −80 °C before extraction of RNA. Three independent experiments were performed.

### 2.2. Confocal Laser Scanning Microscopy Analyses of the Biofilms

*S. xylosus* C2a-B2 biofilms in the Lab-Tek chamber slide system were analyzed by confocal laser scanning microscopy (CLSM) at the three times of incubation: 9, 24 and 48 h. To test for the presence of eDNA in the biofilm, DNase was used under two conditions. First, after incubation at 9, 24 and 48 h and removal of the supernatants, the biofilms were overlaid with DNase solution (100 U, Roche, Mannheim, Germany) for 15 min. Second, DNase (25 U, Roche) was added at the same time as inoculation of the biofilm and the incubation lasted 9, 24 and 48 h. eDNA was stained with 1 µM TOTO-3-iodide (Thermo Fisher Scientific, Molecular probes, IllKirch-Graffenstaden, France) at all incubation times and in all conditions (treated or not with DNase).

After incubation of the biofilms treated or not by DNase, the supernatants in the Lab-Tek chambers were removed and the adhered cells, after washing, were treated with CitiFluor™ (75% AF1 + 25% AF3, UKC Chem. Lab Canterbury, UK) as previously described [33]. The biofilms were observed with a LEICA SP5 CLSM (Leica Microsystems, Nanterre, France, objective x63) at λex = 458 nm to observe the fluorescence of the strain C2a-B2 and at λex = 633 nm to observe DNA stained by TOTO-3. Horizontal cross-sections were acquired consecutively along the *z*-axis using a scanning step size of 1 μm, defining the so-called stacks, to cover the whole biofilm height of one randomly chosen (x, y) coordinate as described [33]. Images were acquired for three biofilm replicates per sampling time. Three-dimensional projections of biofilm structure were reconstructed using the Easy 3D function of the IMARIS 7.0 software (Bitplane, Zurich, Switzerland). Quantitative structural parameters of the biofilms, such as biovolume, substratum coverage, roughness and thickness were calculated using the software COMSTAT 1 [34] under MATLAB.

### 2.3. Phage Induction and Transmission Electron Microscopy

*S. xylosus* C2a-B2 strain was grown for 6 h in BHI up to OD_600_: 0.3–0.4 and was then treated with mitomycin C (2 μg/mL, Sigma-Aldrich, Saint-Quentin-Fallavier, France) to induce prophages. The culture was incubated at 37 °C and 70 rpm for 6 h. Cells were harvested by centrifugation for 10 min at 4500× *g*. The resulting supernatant was filtered through a 0.45 µm membrane and was centrifuged for 30 min at 25,000× *g* and 4 °C. The pellet was used for transmission electron microscopy. The phages were pre-fixed in 0.5% (*w*/*v*) glutaraldehyde, stained with 0.5% (*w*/*v*) uranyl acetate, and examined with a Hitachi H-7650 at a magnification of 120,000-fold.

### 2.4. RNA Extraction, Labeling and Microarray Analyses

For RNA extraction from *S. xylosus* biofilms after 9, 24 or 48 h of incubation, cell pellets were thawed on ice and resuspended in 500 μL of ice-cold Tris-EDTA buffer. Samples were transferred to tubes containing 600 mg of zirconia-silica beads (0.1 mm diameter, BioSpec Products, Bartlesville, OK, USA), 500 μL of acid phenol, 50 µL of sodium dodecyl sulfate (10%) and 3.5 μL of β-Mercaptoethanol. Cells were disrupted using a FastPrep^®^ (MP Biomedicals, Illkirch-Graffenstaden, France). After the addition of 200 μL of chloroform and centrifugation, the aqueous phase containing RNA was collected and purified with the Nucleospin RNA II kit (Macherey Nagel, Hoerdt, France) according to the manufacturer’s instructions. A supplementary treatment was performed with Turbo DNase (Ambion, Austin, TX, USA) to remove residual DNA. The absence of DNA contamination was checked by PCR targeting the *tuf* gene. Total RNA was quantified using a NanoDrop 1000 and RNA quality was analyzed using an Agilent 2100 Bioanalyzer (Agilent Technologies, Santa Clara, CA, USA) according to the manufacturer’s instructions. RNA samples were stored at −80 °C. They were reverse-transcribed to cDNA and labeled as previously described [35]. A complete description of the array developed for *S. xylosus* C2a is available at the NCBI Gene Expression Omnibus (GEO) database under platform accession number GPL19201 and the complete genome of *S. xylosus* C2a is available under accession number LN554884. Significant differences in the probe set intensities between the different conditions were identified using a linear model with an empirical Bayes method using all information probes to moderate the standard errors of the estimated log-fold changes [36]. The probabilities were corrected by the Benjamini–Hochberg procedure to control the false-discovery rate (FDR) with a *p* value cut-off of 0.05. All probes with an FDR ≤ 0.05 are considered to be differentially expressed. Finally, a gene was considered to be differentially expressed if more than 50% of the corresponding probes were differentially expressed and if the ratio of expression was ≥2 or ≤0.5.

The targeted genes for qPCR and primer sequences used to validate microarray data are listed in Supplementary Table S1. The analyses were performed on the same samples of RNA as used for the microarray experiments. The relative fold change of gene expression, using measured *tuf* housekeeping gene expression, was determined by the 2^−ΔΔCt^ method [37].

## 3. Results and Discussion

### 3.1. Evidence of Extracellular DNA by CLSM Analyses

After 9 h, the population in biofilm was 7.2 ± 0.3 log CFU/cm^2^, a small increase to 7.9 ± 0.2 log CFU/cm^2^ was observed at 24 h and then the population remained stable at 8.2 ± 0.5 log CFU/cm^2^ at 48 h.

**Figure 1:**
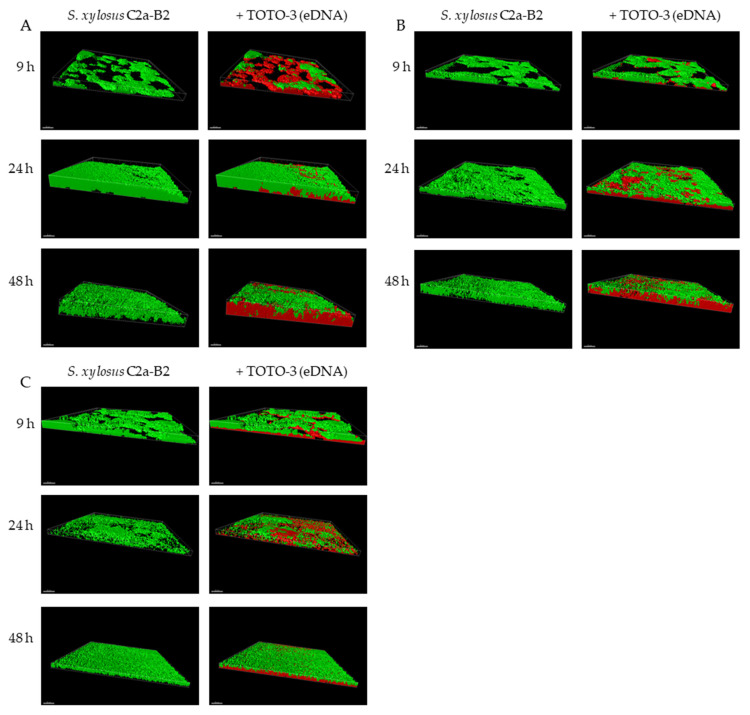
3D projection of biofilms of *S. xylosus* C2a-B2 and DNA stained by TOTO-3 (**A**), after DNase treatment at the end of each incubation time (**B**), after incubation in the presence of DNase from the start (**C**), at three sampling times using Imaris software; Scale bar—30 µm.

**Figure 2:**
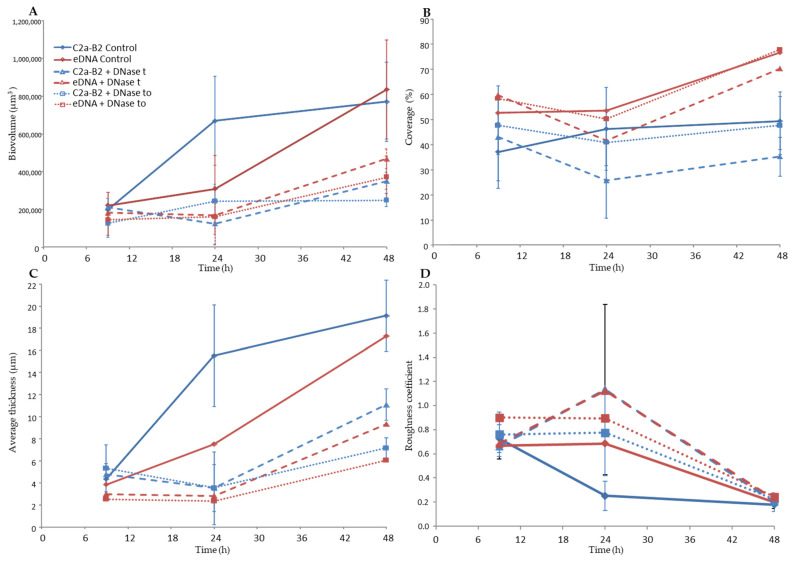
Quantitative structural parameters of the biofilms of *S. xylosus* C2a-B2 and eDNA stained by TOTO-3 at three sampling times. (**A**) Biovolume, (**B**) Coverage, (**C**) Average thickness and (**D**) roughness coefficient. Biovolume C (µm^3^) represented the overall volume of cells in the observation field. Coverage (%) reflected the efficiency of substratum colonization by bacteria. The average thickness (μm) of biofilms was determined from the confocal stack images. Roughness coefficient provided a measure of variations in biofilm thickness and was an indicator of the superficial biofilm interface heterogeneity. Three independent experiments were performed per sampling time. C2a-B2 control (continuous blue curve) and eDNA control (continuous red curve): no treatment; C2a-B2 + DNase t (dashed blue curve) and eDNA + DNase t (dashed red curve): DNase treatment at the end of each incubation time; C2a-B2 + DNase to (dotted blue curve) and eDNA + DNase to (dotted red curve): after incubation in the presence of DNase from the start.

CLSM coupled with cell-impermeant DNA binding fluorescent stain such as TOTO-3 is a powerful tool to study biofilm matrices as it allows real-time visualization of living microorganisms [38]. Using this approach, we observed the architecture of the *S. xylosus* biofilm by recording the fluorescence of the labeled strain *S. xylosus* C2a-B2 (green) and the stained eDNA by TOTO-3 (red) during the 48 h of incubation (Figure 1A). The structural parameters, biovolume, average thickness, roughness and coverage were extracted from confocal images and are represented in Figure 2. *S. xylosus*-B2 produced a rough biofilm composed of aggregates covering 37% of the surface with a maximum thickness of 10.8 µm and an average one of 4.4 µm at 9 h of incubation. Then, a flat thick biofilm was observed from 24 h of incubation up to 48 h. The biovolume and the average thickness of the biofilm increased strongly after 9 h of incubation, while the roughness decreased (Figure 2, C2a control). An increasing amount of eDNA was measured in the *S. xylosus* biofilm during the incubation period, this increase being particularly noticeable between 24 and 48 h, as evidenced by high values of biovolume, thickness and coverage (Figure 1A and Figure 2, eDNA control). Such release of eDNA has been seen in numerous bacteria grown in biofilms such as *S. aureus*, *S. epidermidis*, *Listeria monocytogenes*, *Pseudomonas aeruginosa*, *Bacillus cereus*, and *Burkholderia pseudomallei* [18,19,20,21,39,40,41]. CLSM images revealed eDNA particularly at the base of the biofilm of *S. xylosus* as indicated by the red fluorescence on the bottom while the upper layer displayed green cells (Figure 1A). For *B. pseudomallei*, eDNA was also visualized essentially at the base of the biofilm [41]. For the latter species, as well as for *S. aureus*, eDNA was observed at the beginning of biofilm formation facilitating their adhesion to the support [16,41]. In our study, the first time of observation was 9 h and already a significant amount of eDNA was observed in the *S. xylosus* biofilm (Figure 1A). A previous study has shown that eDNA promoted adhesion of *S. xylosus* cells to hydrophobic surfaces [42].

CLSM images revealed that DNase treatments of *S. xylosus* biofilm decreased eDNA, biovolume and average thickness of the biofilms during the incubation time (Figure 1B,C and, DNase t, DNase t0). The heterogeneity of the biofilms was increased in the presence of DNase as shown by higher roughness coefficient at 24 h of incubation. The use of DNase for biofilm removal is effective, but dependent on the age of the biofilm [21]. Under our conditions, a greater efficiency of DNase was observed on the biofilm of *S. xylosus* at 9 h compared to the following times of 24 and 48 h (Figure 1B,C). Similarly, young biofilms were easily removed, but DNase treatment was less effective on mature biofilm of *S. epidermidis*, *P. aeruginosa* and *B. pseudomallei* [19,22,41]. The addition of DNase at the start of the experiment did not inhibit biofilm formation by *S. xylosus*, but affected its biovolume and average thickness during incubation (Figure 1C and Figure 2, DNase t0). In fact, this treatment at t0 was more effective than that carried out at 48 h, in particular on the thickness of the biofilm at 48 h (Figure 2). DNase inhibited *S. aureus* biofilm formation, detached preformed biofilm and rendered preformed biofilm sensitive to detergent killing [9]. The release of DNA depends on the growing conditions. Under our conditions, the release of DNA by *S. xylosus* was observed during growth in BHI, a nutritionally rich medium. For *L. monocytogenes*, the DNase-sensitive biofilm was only observed in diluted medium with low ionic strength [40]. While for *S. epidermidis*, DNA release has been evidenced in the biofilm formed in whole blood-derived platelet concentrates [18].

### 3.2. Cell Lysis

A transcriptomic approach was used to determine the mechanism involved in the release of DNA. It revealed that 833 (338 down- and 495 up-regulated) genes were differentially expressed at 24 and 48 h, by comparison with 9 h used as reference (Supplementary Table S2). Notably, 458 genes were differentially expressed at the two times of incubation. These genes were classified into different functional categories: the most represented being metabolism (30%) followed by information storage and processing (10%), cellular processes (8%) and phage (7%). This analysis was validated by qPCR on selected genes and a good correlation was noted for the two times of incubation (24 h: r^2^ = 0.91, y = 1.205x − 0.0656; 48 h: r^2^ = 0.93, y = 1.1209x − 0.1745) (Supplementary Table S1).

**Figure 3:**
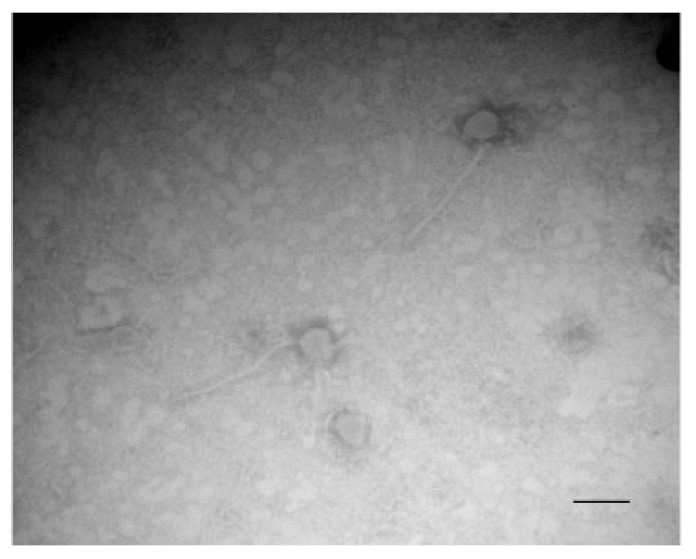
Transmission electron microscopy of phages in the supernatant of *S. xylosus* after induction with mitomycin C. Bar, 100 nm. Magnification, ×120,000.

The transcriptomic data revealed the overexpression of two mechanisms that could be involved in cell lysis, one implicating prophage genes and the other the *cidABC* genes (Table 1). Many prophage genes located in two distinct chromosomal loci were overexpressed (Table 1). The first region of about 15 kb (SXYL_01051-66) is poorly characterized and includes an integrase encoded by SXYL-1051 and several potential tail and host lysis-related genes. The second prophage region of 39.4 kb from SXYL_01727-88 displays the typical organization of the *Siphoviridae* phage genome, with typical functional modules of lysogeny, DNA metabolism, DNA packaging and head, tail and host lysis-related genes [43]. Temperate *Siphoviridae* are the main prophages found in coagulase-negative *Staphylococcus* genomes [44]. The gene SXYL_01783 encodes the phage antirepressor protein. In *Salmonella* phage P22, the antirepressor overcomes the repressor protein involved in the maintenance of lysogeny [45]. In the *S. xylosus* biofilm at both 24 and 48 h incubation, the lysogeny could switch to the lytic cycle. To determine if lytic production of phage particles can be induced from *S. xylosus* C2a, a planktonic culture was treated with mitomycin C Observation by transmission electron microscopy seemed to reveal only one type of phage with a shaped head and a long thin tail, as *Siphoviridae* (Figure 3). A spontaneous release of phages from *S. aureus* biofilm cells has already been observed [31]. These phages were detected over a period of 48–72 h in biofilm cultures. Likewise, lysogenic pneumococcal strains are able to release phage particles during biofilm development by spontaneous induction of prophage and hence release DNA [46]. In *Shewanella oneidensis*, prophage-mediated lysis results in DNA release and enhanced biofilm formation [47]. In this bacterium, induction of the Lambda So prophage occurs in RecA-dependent manner, involving oxidative stress-induced DNA damage as the major trigger. Iron and, in a minor way, H_2_O_2_ are involved in this oxidative stress [48]. For *S. xylosus*, the mechanism that could induce the release of phage particles in the biofilm remains unknown, but we observed that genes involved in iron uptake and response to oxidative stress were up-regulated, as discussed in Section 3.9 and Section 3.10.

**Figure 4:**
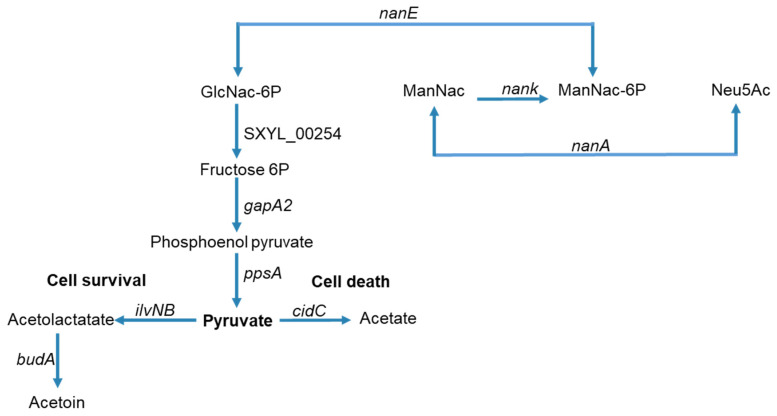
Summary of carbohydrate and amino sugar catabolism *in S. xylosus* biofilm showing the up-regulated genes (the level of expression of these genes and the corresponding enzymes are given in Table 1). GlcNac-6P = *N*-acyl-d-glucosamine 6-phosphate, ManNac = *N*-acyl-d-mannosamine, ManNac-6P = *N*-acyl-d-mannosamine 6-phosphate, Neu5Ac = *N*-acetyl neuraminate.

In addition to overexpression of genes encoding proteins from the two phage loci, the *cidABC* operon in sessile cells of *S. xylosus* was overexpressed particularly at 48 h (>10-fold) (Table 1). This operon has been widely studied in *S. aureus* [26]. CidA forms pores in the membrane allowing the murein hydrolase to reach and degrade peptidoglycan. CidB protein, like CidA, contains multiple predicted membrane-spanning domains, but its role is not yet established. CidC is a pyruvate oxidase that decarboxylates pyruvate to acetate. The *cid* operon plays a significant role during *S. aureus* biofilm development [27]. Thus, the biofilm produced by the *S. aureus cidA* mutant is more loosely compact and less adherent to the substrate. DNase treatment of this biofilm has a small effect, while it destabilizes the wild-type *S. aureus* biofilm revealing that DNA is released because of the *cidA*-mediated lysis of a subset of the bacterial population [27]. In our study, the overexpression of *cidA* from 24 h could have contributed to the lysis of *S. xylosus* and thus the release of DNA. Death and lysis in staphylococcal biofilms are under the control of a regulatory network such as CidR, which induces *cidABC*, *lrgAB* and *alsSD* transcription in response to the accumulation of intracellular pyruvate or acetate [24,26]. In our conditions, *cidABC*, as already mentioned, was overexpressed and *lrgAB* was not modulated. The *alsSD* operon is not present in the genome of *S. xylosus*, but *ilvNB* encoding an acetolactate synthase and *budA* encoding an acetolactate decarboxylase leading to acetoin were overexpressed (Table 1). In *S. aureus* biofilm, acetate derived from CidC activity potentiates cell death by a mechanism dependent on intracellular acidification and respiratory inhibition and AlsSD counters CidC by diverting carbon flux towards neutral rather than acidic byproducts, consuming protons in the process [49]. Based on our results, and as summarized in Figure 4 for *S. xylosus*, CidC, IlvNB, and BudA could modulate cell death to achieve optimal biofilm biomass. Lysis might be an advantage for the biofilm community because the remaining cells can gain nutrients from dead and lysed neighboring cells, as we will discuss in the section on metabolism.

### 3.3. Slow Cellular Process in the Mature Biofilm

The physiology of *S. xylosus* biofilm, like *S. aureus* and *S. epidermis* biofilms, is characterized by a general down-regulation of active cell processes such as protein, DNA, and cell wall syntheses that is typical of slow-growing cells [50]. However, several genes involved in the DNA machinery were up-regulated (Table 1). Thirteen genes encoding transcriptional regulators orchestrating gene activity and 12 genes involved in translation and ribosomal biogenesis were up-regulated. It is noteworthy that, *walK* was up-regulated (Table 1). It encodes a member of the two-component regulatory system WalRK. In *S. aureus*, WalRK positively controls biofilm formation [51] and is essential for cell viability mainly by controlling the transcription of cell wall lysing enzymes [52]. WalKR in *S. aureus* activated the transcription of nine genes involved in the different steps of cell wall turnover (*lytM*, *atlA*, *isaA*, *sceD*, *ssaA*, and four *ssaA*-related genes) [51]. In our study, only the *isaA* gene, and two *sceD* genes were up-regulated (Table 1).

### 3.4. DNA/RNA, Protein Repair Systems

It is worthy of mention that, 10 genes encoding proteins involved in DNA/RNA repair were up-regulated in *S. xylosus* in biofilm (Table 1). Among them, *dinB* encodes DNA polymerase IV, which is up-regulated during the SOS response to DNA damage in *Escherichia coli* and contributes to spontaneous mutation in slow-growing or non-growing cells [53]. The *recF* gene, which was up-regulated in our conditions, encodes RecF, which is required for DNA replication and for daughter strand gap repair [54]. This gene is also up-regulated in *S. epidermidis* in biofilm [55]. The gene *tag* encoding DNA-3-methyladenine glycosylase was up-regulated in *S. xylosus*; this enzyme is a base excision repair glycosylase that recognizes and excises a variety of alkylated bases from DNA [56]. The gene SXYL_01206 encoding the RadC family protein was up-regulated in *S. xylosus*. This protein is involved in DNA replication and repair [57]. The gene *rnr* encoding a ribonuclease R was also up-regulated. In *E. coli*, this RNase acts over a range of substrates, such as, ribosomal, transfer, messenger, and small non-coding RNAs [58]. The UvrABC repair system, encoded by *uvrABC*, which was overexpressed in our conditions, catalyzes the recognition and processing of DNA lesions. Endonuclease III is a ubiquitous DNA repair enzyme that repairs oxidized pyrimidine base lesions in DNA. It is encoded by the SXYL_02241 gene, which was up-regulated in our conditions.

Bacterial cells are equipped to adapt to various environmental conditions. They have developed a general response, like heat shock proteins, chaperones and ATP-dependent proteases, to deal with damaged proteins. In our study, 10 genes encoding proteases and chaperones involved in protein turnover were up-regulated (Table 1). *S. xylosus* overexpressed *ctsR* and the *clpC*, *clpB*, *dnaK* and *groES* genes. These genes are identified in *S. aureus* as belonging to the CtsR regulon [59]. Moreover, *dnaK* belongs to a cluster, which includes *grpE*, *dnaJ* and *hcrA* encoding a transcriptional regulator. All these genes were up-regulated in *S. xylosus* in biofilm. In *S. aureus*, the *dnaK* and *groES* operons also belong to the HrcA regulon embedded within the CtsR regulon, which controls HrcA synthesis [59]. A similar transcriptional regulation could occur in *S. xylosus* as revealed by the network of genes up-regulated under our conditions. The gene *mecA* up-regulated by *S. xylosus* encodes MecA, which enables the recognition and targeting of unfolded and aggregated proteins to the ClpC protease. Finally, the gene SXL_00505, up-regulated, encodes a YidC/Oxa1 family membrane protein insertase. These family members can function depending on the context as insertases, chaperones, and assembly factors for transmembrane proteins [60].

### 3.5. Pyrimidine and Purine Salvage

Uracil arising from cell lysis could be used as pyrimidine source. It can be taken up by the *pyrP* encoded permease, which is a part of the *pyr* operon including *pyrCBPR*, all of which was highly up-regulated at 48 h of incubation in *S. xylosus* (Table 1). PyrR can act as a phosphoribosyltransferase leading to UMP and is a regulatory protein of the *pyr* operon in *Bacillus subtilis* [61]. The genes *pyrCB*, *carAB*, and *glnA2* genes, which were all up-regulated, could also contribute to the synthesis of UMP from glutamate/glutamine as already described for *S. xylosus* [62] (Table 1). As for *S. xylosus*, the genes encoding pyrimidine were strongly up-regulated in *S. aureus* in biofilm compared to planktonic cultures [63]. The *nrdEF* genes up-regulated in *S. xylosus* in biofilm could participate in the synthesis of pyrimidine deoxynucleotides. Three genes, including the repressor *purR*, which is involved in purine synthesis were up-regulated (Table 1). In *B. subtilis*, PurR regulated the 12-gene *pur* operon required for de novo synthesis of purine from the IMP pathway [61]. Such regulation could happen in *S. xylosus*; this operon is also down-regulated in *S. aureus* grown in biofilm [63].

### 3.6. Amino Sugar Catabolism 

One of the main features of *S. xylosus* in sessile conditions was its potential to use amino sugars released from the cell wall of lysed bacteria as a carbon source. These amino sugars are catabolized via the amino sugar pathway, for which a cluster of four genes was up-regulated (*nan*, SXYL_00403-406, Table 1 and Figure 4). A complete *nan* system was defined as one that minimally includes orthologues of genes encoding NanA, NanE, and NanK [64]. This is the case with *S. xylosus*, which also comprises a transcriptional regulator of the RpiR family (Table 1). The *nan* systems of *E. coli* and *S. aureus* also include a regulator, albeit very different from each other and not characterized for *S. aureus* [64]. This *nan* pathway led to N-acetyl-glucosamine-6-phosphate, which could be further catabolized to pyruvate via several steps (Figure 4). In addition, in *S. xylosus* in biofilm, four genes involved in the pentose and glucuronate pathways were overexpressed (Table 1). Pyruvate could be catabolized by enzymes encoded by *cidC*, *budA* and *ilvNB* as described above (Figure 4) but all other genes involved in its catabolism and in the TCA cycle were down-regulated (Table S2). Finally, two *cydBA* genes encoding cytochrome oxidase, which generates a proton motive force, were overexpressed (Table 1). In *E. coli*, cytochrome bd oxidase was expressed under O_2_-limited conditions [65]. This suggests that *S. xylosus* embedded in an eDNA matrix as observed by microscopy seems to perceive an anaerobic environment as revealed by the transcriptomic data related to the catabolism of sugars.

### 3.7. Amino Acid Synthesis

Amino acids could be released during cell lysis. Indeed, two clusters of genes (SXYL_00264-66; SXYL_00661-65) coding for ABC type amino acid transporters and one gene encoding an ammonia permease were up-regulated in *S. xylosus* in biofilm, particularly at 48 h of incubation (Table 1). One of these clusters was involved in the transport of glutamate, a key component as the main nitrogen donor. In addition, glutamate could be synthesized by two pathways. One could involve alpha ketoglutarate and glutamine catabolized by glutamate synthase encoded by *gltDB*, which was overexpressed, together with *gltC* coding for a transcriptional activator (Table 1). In *B. subtilis*, the main regulatory role of GltC appears to be the prevention of a cycle of simultaneous glutamate synthesis and degradation [66]. The other pathway could involve the degradation of histidine with the overexpression of *hutH* and *hutUI* encoding histidine catabolic enzymes (Table 1). Then glutamate could be catabolized to glutamine-by-glutamine synthases encoded by *glnA1*, which was overexpressed only at 48 h of incubation, and *glnA2*, which was overexpressed at 24 and 48 h (Table 1). The *glnA1* gene is in an operon with *glnR* encoding a repressor. In *B. subtilis*, GlnR represses the *gln* operon in the presence of glutamine. This repression is considered as a fine-tuning mechanism of *gln* expression [66]. Finally, glutamine could be involved in the synthesis of pyrimidine, as described in Section 3.5.

Almost all genes involved in the synthesis of branched-chain amino acids were up-regulated in *S. xylosus* in biofilm, especially at 48 h of incubation (cluster SXYl_00867-74, Table 1). Several genes involved in the synthesis of glycine, and of cysteine/methionine and alanine/lysine were up-regulated. Noteworthily, *alr2* was highly overexpressed. It encodes an alanine racemase, which furnishes d-alanine for the synthesis of the cell wall. Surprisingly, the gene *ldhD* encoding a d-lactate dehydrogenase was highly up-regulated (>10-fold) both at 24 and 48 h. d-lactate instead of the usual d-alanine could be introduced in peptidoglycan, as already reported for *Staphylococcus haemolyticus* resistant to vancomycin [67].

### 3.8. Cofactor, Vitamin Synthesis

*S. xylosus* in biofilm overexpressed the cluster *rib* involved in riboflavin synthesis (Table 1). It also overexpressed a cluster of two genes encoding energy-coupling factor transporters (Table 1). These transporters mediate the uptake of essential vitamins and metal ions in many prokaryotes, in particular for those bacteria lacking the pathways for folate, biotin, and thiamine biosynthesis [68]. Biotin and thiamine are two vitamins essential for *S. xylosus* growth [69]. Seven other overexpressed genes are involved in the synthesis of porphyrin, cofactor and folate (Table 1).

### 3.9. Inorganic Ion Transport

In our conditions, three genes of the cluster *pstSCAB* involved in the import of inorganic phosphate were up-regulated at 48 h of incubation (Table 1). In *S. aureus*, the two-component system PhoPR is required for the expression of *pstSCAB* and is necessary for its growth under phosphate limiting conditions [70]. In our conditions, the genes encoding this system were down-regulated (Table S2). In *S. xylosus* biofilm, phosphate seems not to be limiting, as DNA was released in the growth medium.

Iron, an essential cofactor for several enzymes, is complexed with different proteins and its concentration is finely regulated. Fur (ferric uptake regulator) is involved in iron homeostasis and, is a repressor of three iron-acquiring systems (*hts*, *sst*, *fhu*) in *S. xylosus* [36,62]. In biofilm, *fur* was up-regulated, and consequently, these three systems were under-expressed (Table S2). Two clusters of genes, *sitABC* and SXYL_00561-63, were overexpressed at 24 and 48 h of incubation. The cluster *sitABC*, encoding an iron-regulated ABC transport involved in divalent metal uptake, was modulated in the presence of ferrous iron (FeSO4), while the cluster SXYL_00561-63 was highly up-regulated in the presence of ferritin in *S. xylosus* [71]. Note that, the gene *dps* encoding a Dps family protein was up-regulated. The crystal structure of Dps reveals structural homology with ferritins, a large family of iron storage proteins [72]. This led us to suppose that the cluster SXYL_00561-63 could be involved in the uptake of iron from Dps in *S. xylosus* in biofilm.

As mentioned for iron, all metal ions are required for biological reactions, but they are toxic in excess, and the intracellular availability of each is tightly regulated [73]. *S. xylosus* in biofilm up-regulated three genes involved in manganese acquisition and two clusters involved in metal efflux, one for zinc and cobalt and the other for copper (Table 1). Surplus of zinc and cobalt can be sensed by the regulator CzrA, encoded by *czrA*, which was highly up-regulated in our conditions, inducing the metal-efflux protein encoded by SXYL_00783. The copper chaperone CopZ, encoded by *copZ*, which was highly up-regulated in our conditions, could lead to the transcriptional de-repression of *copA*, which could result in the export of copper [73].

### 3.10. Response to Stress

The transcriptome profile of *S. xylosus* revealed the activation of stress-induced pathways within biofilm. The up-regulation of the *cudTCAbetA* cluster encoding a choline transporter (CudT), a regulator (CudC) and two enzymes (CudA, BetA) to form glycine betaine a powerful osmoprotectant indicated the perception of an osmotic stress (Table 1). In *S. xylosus*, the *cudAbetA* genes are up-regulated by choline and elevated NaCl concentrations [74]. In addition, the cluster *mnhF2-A2* and three genes encoding Na^+^/H^+^ antiporter systems were up-regulated, particularly at 48 h of incubation. Similarly, the genes *opuD* encoding a glycine betaine transporter, *prop* a proline betaine transporter and *mnhA* a Na^+^/H^+^ antiporter unit are up-regulated in *S. aureus* in biofilm [75]. In addition, both genes encoding a glycine betaine transporter and a glycine betaine aldehyde dehydrogenase are up-regulated in *S. epidermidis* in biofilm [55]. 

*S. xylosus* also had to cope with oxidative stress, as shown by the up-regulation of seven genes involved in detoxification. Five of these genes (*katB*, *trxB*, *ahpCF*, *SXL_00895*) are under the control of the repressor PerR, whose gene is not modulated in our conditions (Table 1). These genes are overexpressed following nitrosative stress in a meat model [35]. In addition, *katA* was down-regulated, and *sodA* was not modulated in *S. xylosus* in biofilm, whereas these genes were found under-expressed in *S. aureus* in biofilm versus stationary growth phase [63]. A response to oxidative stress has already been reported for several biofilm-forming bacteria [76]. 

## 4. Conclusions

This study provides data on the physiology of *S. xylosus* in biofilm. It reveals that eDNA is a major component of the extracellular polymeric matrix and can be released by two mechanisms of cell lysis, lytic phage and the CidABC system. This lysis could furnish nutrients such as amino sugars, amino acids, nucleotides, and ions for the remaining cells. *S. xylosus* has developed defense mechanisms against osmotic and oxidant stresses. In addition, *S. xylosus* overexpressed several genes involved in DNA/RNA repair systems and in protein turnover. The ability of *S. xylosus* to form biofilm embedded in eDNA matrix allows it to colonize and survive in manufacturing environment.

## Figures and Tables

**Table 1:** Genes of *S. xylosus* discussed in this study, overexpressed at 24 h and/or 48 h compared to 9 h in biofilm.

			Mean Ratio of Expression	
Gene ID	Gene Name	Description	24 h/9 h	48 h/9 h
**CELL LYSIS**				
SXYL_01051		Phage integrase	7.2	17.9
SXYL_01054-66		Phage proteins	5.7 *	12.4 *
SXYL_01727-84		Phage proteins	3.8 *	3.1 *
SXYL_00365-66	*cidAB*	Holin-like protein CidA and CidB	2.7 *	13.5 *
SXYL_00367	*cidC*	Pyruvate oxidase		9.2
SXYL_00431	*budA*	Alpha-acetolactate decarboxylase	8.3	4.9
SXYL_00873-74	*ilvNB*	Acetolactate synthase		2.1 *
**INFORMATION STORAGE, PROCESSING, CELLULAR PROCESSES**				
**Replication, recombination**				
SXYL_01294	*dnaG*	DNA primase	2.2	2.1
SXYL_00005-06	*gyrBA*	DNA gyrase subunits B,A	2.1 *	
**Transcription**				
SXYL_00212		Transcriptional regulator	2.1	2.6
SXYL_00418	*marR*	MarR-family transcriptional regulator	4.1	4.5
SXYL_00690		MarR family transcriptional regulator	3.2	5.1
SXYL_00457		Acetyltransferase	2.5	
SXYL_00523		PadR-like family transcriptional regulator	5.9	7.0
SXYL_00786		Transcriptional regulator, GntR family	5.1	
SXYL_00904		Transcriptional regulator, GntR family		2.3
SXYL_02403		Transcriptional regulator, GntR family	4.3	5.8
SXYL_01239	*mnmA*	tRNA-specific 2-thiouridylase MnmA	2.4	2.8
SXYL_01352		AraC-family transcriptional regulator	2.3	
SXYL_02345		LacI-family transcriptional regulator	2.3	2.1
SXYL_02663		Transcriptional regulator, LacI family		2.3
SXYL_02482		Transcriptional regulator, MerR family		2.1
SXYL_02549		Transcriptional regulator	5.1	5.1
SXYL_02596		HxlR family transcriptional regulator	3.1	
SXYL_00022	*walK*	Sensor protein kinase walK	2.1	
SXYL_00323	*isaA*	Probable transglycosylase IsaA		2.8
SXYL_00116	*sceD2*	Probable transglycosylase SceD 2	8.7	18.6
SXYL_00117	*sceD1*	Probable transglycosylase SceD1	13.7	21.7
**Translation, ribosomal biogenesis**				
SXYL_01279-80	*prmA*	Ribosomal protein L11 methyltransferase	3.6 *	3.5 *
SXYL_01281	*rpsU*	30S ribosomal protein S21	2.1	2.0
SXYL_01549	*rpsN*	30S ribosomal protein S14	3.5	3.5
SXYL_01550	*rpmG2*	50S ribosomal protein L33 2	2.0	
SXYL_01615	*infB*	Translation initiation factor IF-2	2.0	
SXYL_01619	*rimP*	Ribosome maturation factor RimP		2.2
SXYL_01673		Peptide deformylase-like		2.1
SXYL_02139	*queC*	7-cyano-7-deazaguanine synthase	3.2	3.3
SXYL_02689	*rsmG*	Ribosomal RNA small subunit methyltransferase G	2.8	2.5
SXYL_02690-91	*mnmGE*	tRNA uridine 5-carboxymethylaminomethyl, GTPase	2.9 *	2.6 *
**DNA/RNA repair**				
SXYL_00942	*dinB*	DNA polymerase IV	2.8	3.7
SXYL_00004	*recF*	DNA replication and repair protein RecF	2.1	
SXYL_01201	*tag*	DNA-3-methyladenine glycosylase	2.1	
SXYL_01206		DNA repair RadC family protein		2.8
SXYL_02062	*rnr*	Ribonuclease R		2.1
SXYL_01796	*uvrC*	UvrABC system protein C	3.7	2.1
SXYL_02088-89	*uvrAB*	UvrABC system protein A, B	4.3 *	3.5 *
SXYL_00943		Putative exonuclease	2.3	3.0
SXYL_02241		Endonuclease III	2.2	
**Protein turnover**				
SXYL_02399	*ctsR*	Transcriptional regulator CtsR	5.4	6.1
SXYL_02396	*clpC*	ATP-dependent Clp protease ATP-binding subunit ClpC	4.9	4.8
SXYL_01946	*clpB*	Chaperone protein ClpB	10.8	13.4
SXYL_00898	*groS*	10 kDa chaperonin	2.1	
SXYL_01275	*hrcA*	Heat-inducible transcription repressor HrcA	3.8	4.6
SXYL_01276	*grpE*	Protein GrpE	3.9	4.8
SXYL_01277	*dnaK*	Chaperone protein DnaK	3.4	3.7
SXYL_01278	*dnaJ*	Chaperone protein dnaJ	3.9	3.9
SXYL_01192	*clpX*	ATP-dependent Clp protease ATP-binding subunit ClpX	2.2	2.1
SXYL_01933	*mecA*	Adapter protein MecA	2.0	
SXYL_02418	*hslO*	33 kDa chaperonin	2.5	2.1
SXYL_00548		Heat shock protein Hsp20	7.6	7.3
SXYL_00549		Heat shock protein Hsp20	9.5	8.8
SXYL_00505		Membrane protein insertase, YidC/Oxa1 family	2.2	
**PYRIMIDINE, PURINES SALVAGE**				
**Pyrimidine**				
SXYL_00796	*pdp*	Pyrimidine-nucleoside phosphorylase	2.1	
SXYL_01688-91	*pyrCBPR*	Pyrimidine synthesis		8.2 *
SXYL_01407		Nucleoside-diphosphate-sugar epimerase	2.1	
SXYL_02117-18	*nrdFE*	Ribonucleoside-diphosphate reductase		2.8 *
**Purine**				
SXYL_00017	*purA*	Adenylosuccinate synthetase		2.2
SXYL_01548	*guaC*	GMP reductase	2.5	3.0
SXYL_02435	*purR*	Pur operon repressor	2.0	
**CARBOHYDRATE CATABOLISM**				
**Amino sugar catabolism**				
SXYL_00403	*nanE*	*N*-acetylmannosamine-6-phosphate 2-epimerase	2.5	
SXYL_00404		RpiR family transcriptional regulator	2.1	
SXYL_00405	*nanK*	*N*-acetylmannosamine kinase	2.5	
SXYL_00406	*nanA*	*N*-acetylneuraminate lyase	3.9	2.7
SXYL_00254		Glucosamine-6-phosphate deaminase	3.4	
**Glycolysis**				
SXYL_00773-76	*mtlDFA*	PTS system mannitol	4.1 *	5.4 *
SXYL_00253		PTS system, glucose-specific IIBC component	4.7	2.7
SXYL_01179	*gapA2*	Glyceraldehyde-3-phosphate dehydrogenase 2	2.7	
SXYL_00207	*ppsA*	Pyruvate phosphate dikinase	5.6	5.6
SXYL_00208		Putative pyruvate, phosphate dikinase regulatory protein	6.3	7.3
**Pentose, glucuronate**				
SXYL_01454		Xylose isomerase-like protein		2.3
SXYL_00123	*araB*	l-ribulokinase	2.1	
SXYL_00124	*araD*	l-ribulose-5-phosphate 4-epimerase	2.2	2.4
SXYL_02343		2-keto-3-deoxygluconate kinase	2.6	5.4
**Energy**				
SXYL_01849-50	*cydBA*	Cytochrome bd-type quinol oxidase	2.5 *	
**AMINO ACID METABOLISM**				
**Transport**				
SXYL_00264-66		Amino acid ABC transporter		4.1 *
SXYL_02464		Ammonia permease	2.7	4.3
SXYL_00661-65		ABC-type amino acid transport system, Glutamate ABC transporter		2.7 *
**Glutamate/glutamine**				
SXYL_02459	*gltD*	NADH-glutamate synthase small unit	2.4	
SXYL_02460	*gltB*	Glutamate synthase large subunit	2.9	2.3
SXYL_02461	*gltC*	Transcription activator of glutamate synthase operon		3.6
SXYL_01568-69	*glnA1R*	Glutamine synthetase, repressor		3.4 *
SXYL_00107-08	*glnA2*	Glutamine synthetase, Aldehyde dehydrogenase	6.7 *	4.5 *
SXYL_01686-87	*carBA*	Carbamoyl-phosphate synthase		4.1 *
**Histidine**				
SXYL_00008	*hutH*	Histidine ammonia-lyase (Histidase)	6.4	5.0
SXYL_00617-18	*hutUI*	Urocanate hydratase, Imidazolonepropionase	2.9 *	3.1 *
**Valine/leucine/isoleucine**				
SXYL_00867-74	*ilvADCBACNB*	Valine, leucine, isoleucine synthesis		2.7 *
**Glycine/serine/threonine**				
SXYL_01317-19	*gcvTPAPB*	Aminomethyltransferase, probable glycine dehydrogenase	3.0 *	2.3 *
**Cysteine/methionine**				
SXYL_02417	*cysK*	Cysteine synthase		2.3
SXYL_02636-37	*cysIJ*	Sulfite reductase (NADPH) hemoprotein		3.3 *
SXYL_01238		Putative cysteine desulfurase	2.5	3.2
SXYL_01672	*fmt*	Methionyl-tRNA formyltransferase		2.2
**Alanine/lysine**				
SXYL_01473	*lysA*	Diaminopimelate decarboxylase	5.3	3.3
SXYL_01474	*alr2*	Alanine racemase 2	11.9	7.6
SXYL_01475		Uncharacterized hydrolase	12.1	7.6
SXYL_01476-78	*dapHBA*	2,3,4,5-tetrahydropyridine-2,6-dicarboxylate *N*-acetyltransferase,	4.1 *	2.5 *
		4-hydroxy-tetrahydrodipicolinate reductase, synthase		
SXYL_02665		Dihydrodipicolinate synthase	2.0	3.2
SXYL_00325	*ldhD*	d-lactate dehydrogenase	10.3	11.9
**COFACTOR, VITAMIN SYNTHESIS**				
SXYL_01097-100	*ribDEBAH*	Riboflavin biosynthesis	2.8 *	3.7 *
SXYL_00734-35	*ecfA2T*	Energy-coupling factor transporter	2.2 *	
SXYL_01194	*hemA*	Glutamyl-tRNA reductase	2.0	
SXYL_01196	*hemC*	Porphobilinogen deaminase	2.5	
SXYL_02635	*cobA*	Uroporphyrin-III C-methyltransferase	2.0	2.9
SXYL_00839	*thiD*	Hydroxymethylpyrimidine/phosphomethylpyrimidine kinase	2.0	
SXYL_01231		HesA/MoeB/ThiF family protein	2.1	2.0
SXYL_01893	*menF*	Isochorismate synthase	2.0	2.4
SXYL_02295	*folE2*	GTP cyclohydrolase FolE2	2.2	
**INORGANIC ION TRANSPORT**				
**Phosphate**				
SXYL_01484-86	*pstSCA*	ABC-type phosphate transport system		2.4 *
**Fe**				
SXYL_01359	*fur*	Ferric uptake regulation protein	2.3	2.1
SXYL_02216-18	*sitABC*	ABC metal ion transport system, Iron/manganese/zinc	2.0 *	4.7 *
SXYL_00561-63		Oxidoreductase, Monooxygenase, Transporter	5.6 *	12.4 *
SXYL_00793	*dps*	Dps family protein	3.6	3.7
**Mn, Co, Zn, Cu**				
SXYL_02659-60	*mtsC*	Metal ion ABC transporter, manganese		3.4 *
SXYL_00416		Putative ABC-type Mn Zn transport system periplasmic	2.2	
SXYL_00783-84	*czrA*	Co Zn Cd efflux system component, Zn, Co transport repressor CzrA	11.6 *	11.8 *
SXYL_00326-27	*copZA*	Copper chaperone CopZ, Copper-exporting P-type ATPase A	10.0 *	13.2 *
SXYL_00512		Putative cation efflux family protein	4.5	3.4
**RESPONSE TO STRESS**				
**Osmotic**				
SXYL_00223	*cudT*	Choline transporter		2.4
SXYL_00224	*cudC*	Putative transcriptional regulator	4.9	5.6
SXYL_00225	*cudA*	Glycine betaine aldehyde dehydrogenase	21.1	21.4
SXYL_00226	*betA*	Oxygen-dependent choline dehydrogenase	13.4	12.3
SXYL_02221-26	*mnhF2E2D2C2B2A2*	Na(+)/H(+) antiporter		2.7 *
SXYL_02219		Putative NhaP-type Na+ H+ and K+ H+ antiporter		2.1
SXYL_00425		Na(+)/H(+) exchanger		2.1
SXYL_00407		Sodium:solute symporter family protein	4.0	2.3
**Oxidative**				
SXYL_01551	*katB*	Catalase B		2.4
SXYL_02505	*katA*	Catalase A	4.2	5.7
SXYL_01797	*trxA*	Thioredoxin	2.6	
SXYL_02083	*trxB*	Thioredoxin reductase	2.3	2.3
SXYL_02534-35	*ahpCF*	Alkyl hydroperoxide reductase	4.2 *	4.2 *
SXYL_00895		Nitroreductase family protein	4.9	4.1

* Means of the expression of the clustered genes overexpressed.

## Data Availability

Not applicable.

## Supplementary Materials

The following are available online at https://www.mdpi.com/article/10.3390/microorganisms9112192/s1, Table S1: Targeted genes of *Staphylococcus xylosus* for the validation of microarray data by qPCR: expression at 24 h and 48 h in biofilm compared with 9 h. Table S2: Total genes of *Staphylococcus xylosus* differentially expressed at 24 h and/or 48 h of incubation compared to 9 h in biofilm.

## References

[1] Nagase N., Sasaki A., Yamashita K., Shimizu A., Wakita Y., Kitai S., Kawano J. (2002). Isolation and species distribution of staphylococci from animal and human skin. J. Vet. Med. Sci..

[2] Verdier-Metz I., Gagne G., Bornes S., Monsallier F., Veisseire P., Delbès-Paus C., Montel M.C. (2012). Cow teat skin, a potential source of diverse microbial populations for cheese production. Appl. Environ. Microbiol..

[3] Delbès C., Ali-Mandjee L., Montel M.C. (2007). Monitoring bacterial communities in raw milk and cheese by culture-dependent and -independent 16S rRNA gene-based analyses. Appl. Environ. Microbiol..

[4] Leroy S., Giammarinaro P., Chacornac J.P., Lebert I., Talon R. (2010). Biodiversity of indigenous staphylococci of naturally fermented dry sausages and manufacturing environments of small-scale processing units. Food Microbiol..

[5] Planchon S., Gaillard-Martinie B., Dordet-Frisoni E., Bellon-Fontaine M.N., Leroy S., Labadie J., Hébraud M., Talon R. (2006). Formation of biofilm by *Staphylococcus xylosus*. Int. J. Food Microbiol..

[6] Götz F. (2002). *Staphylococcus* and biofilms. Mol. Microbiol..

[7] Branda S.S., Vik A., Friedman L., Kolter R. (2005). Biofilms: The matrix revisited. Trends Microbiol..

[8] Moormeier D.E., Bayles K.W. (2017). *Staphylococcus aureus* biofilm: A complex developmental organism. Mol. Microbiol..

[9] Izano E.A., Amarante M.A., Kher W.B., Kaplan J.B. (2008). Differential roles of poly-N-acetylglucosamine surface polysaccharide and extracellular DNA in *Staphylococcus aureus* and *Staphylococcus epidermidis* biofilms. Appl. Environ. Microbiol..

[10] Arciola C.R., Campoccia D., Ravaioli S., Montanaro L. (2015). Polysaccharide intercellular adhesin in biofilm: Structural and regulatory aspects. Front. Cell. Infect. Microbiol..

[11] Planchon S., Desvaux M., Chafsey I., Chambon C., Leroy S., Hébraud M., Talon R. (2009). Comparative subproteome analyses of planktonic and sessile *Staphylococcus xylosus* C2a: New insight in cell physiology of a coagulase-negative *Staphylococcus* in biofilm. J. Proteome Res..

[12] Hussain C., Herrmann M., von Eiff C., Perdreau-Remington F., Peters G. (1997). A 140-kilodalton extracellular protein is essential for the accumulation of *Staphylococcus epidermidis* strains on surfaces. Infect. Immun..

[13] Cucarella C., Tormo M.A., Ubeda C., Trotonda M.P., Monzón M., Peris C., Amorena B., Lasa I., Penadès J.R. (2004). Role of biofilm-associated protein bap in the pathogenesis of bovine *Staphylococcus aureus*. Infect. Immun..

[14] Gross M., Cramton S.E., Götz F., Peschel A. (2001). Key role of teichoic acid net charge in *Staphylococcus aureus* colonization of artificial surfaces. Infect. Immun..

[15] Sadovskaya I., Vinogradov E., Li J., Jabbouri S. (2004). Structural elucidation of the extracellular and cell-wall teichoic acids of *Staphylococcus epidermidis* RP62A, a reference biofilm-positive strain. Carbohydr. Res..

[16] Mann E.E., Rice K.C., Boles B.R., Endres J.L., Ranjit D., Chandramohan L., Tsang L.H., Smeltzer M.S., Horswill A.R., Bayles K.W. (2009). Modulation of eDNA release and degradation affects *Staphylococcus aureus* biofilm maturation. PLoS ONE.

[17] Kavanaugh J.S., Flack C.E., Lister J., Ricker E.B., Ibberson C.B., Jenul C., Moormeier D.E., Delmain E.A., Bayles K.W., Horswill A.R. (2019). Identification of extracellular DNA-binding proteins in the biofilm matrix. mBio.

[18] Loza-Correa M., Ayala J.A., Perelman I., Hubbard K., Kalab M., Yi Q.L., Taha M., de Pedro M.A., Ramirez-Arcos S. (2019). The peptidoglycan and biofilm matrix of *Staphylococcus epidermidis* undergo structural changes when exposed to human platelets. PLoS ONE.

[19] Whitchurch C.B., Tolker-Nielsen T., Ragas P.C., Mattick J.S. (2002). Extracellular DNA required for bacterial biofilm formation. Science.

[20] Jakubovics N.S., Shields R.C., Rajarajan N., Burgess J.G. (2013). Life after death: The critical role of extracellular DNA in microbial biofilms. Lett. Appl. Microbiol..

[21] Okshevsky M., Regina V.R., Meyer R.L. (2015). Extracellular DNA as a target for biofilm control. Curr. Opin. Biotechnol..

[22] Qin Z., Ou Y., Yang L., Zhu Y., Tolker-Nielsen T., Molin S., Qu D. (2007). Role of autolysin-mediated DNA release in biofilm formation of *Staphylococcus epidermidis*. Microbiology.

[23] Bose J.L., Lehman M.K., Fey P.D., Bayles K.W. (2012). Contribution of the *Staphylococcus aureus* Atl AM and GL Murein Hydrolase Activities in Cell Division, Autolysis, and Biofilm Formation. PLoS ONE.

[24] Sadykov M.R., Bayles K.W. (2012). The control of death and lysis in staphylococcal biofilms: A coordination of physiological signals. Curr. Opin. Microbiol..

[25] Groicher K.H., Firek B.A., Fujimoto D.F., Bayles K.W. (2000). The *Staphylococcus aureus lrgAB* operon modulates murein hydrolase activity and penicillin tolerance. J. Bacteriol..

[26] Rice K.C., Bayles K.W. (2008). Molecular control of bacterial death and lysis. Microbiol. Mol. Biol. Rev..

[27] Rice K.C., Mann E.E., Endres J.L., Weiss E.C., Cassat J.E., Smeltzer M.S., Bayles K.W. (2007). The cidA murein hydrolase regulator contributes to DNA release and biofilm development in *Staphylococcus aureus*. Proc. Natl. Acad. Sci. USA.

[28] Yang S.J., Dunman P.M., Projan S.J., Bayles K.W. (2006). Characterization of the *Staphylococcus aureus* CidR regulon: Elucidation of a novel role for acetoin metabolism in cell death and lysis. Mol. Microbiol..

[29] Webb J.S., Givskovy M., Kjelleberg S. (2003). Bacterial biofilms: Prokaryotic adventures in multicellularity. Curr. Opin. Microbiol..

[30] Webb J.S., Lau M., Kjelleberg S. (2004). Bacteriophage and phenotypic variation in *Pseudomonas* aeruginosa biofilm development. J. Bacteriol..

[31] Resch A., Fehrenbacher B., Eisele K., Schaller M., Götz F. (2005). Phage release from biofilm and planktonic *Staphylococcus aureus* cells. FEMS Microbiol. Lett..

[32] Vermassen A., Dordet-Frisoni E., de La Foye A., Micheau P., Laroute V., Leroy S., Talon R. (2016). Adaptation of *Staphylococcus xylosus* to nutrients and osmotic stress in a salted meat model. Front. Microbiol..

[33] Leroy S., Lebert I., Andant C., Talon R. (2020). Interaction in dual species biofilms between *Staphylococcus xylosus* and *Staphylococcus aureus*. Int. J. Food Microbiol..

[34] Heydorn A., Nielsen A.T., Hentzer M., Sternberg C., Givskov M., Ersbøll B.K., Molin S. (2000). Quantification of biofilm structures by the novel computer program COMSTAT. Microbiology.

[35] Vermassen A., de la Foye A., Loux V., Talon R., Leroy S. (2014). Transcriptomic analysis of *Staphylococcus xylosus* in the presence of nitrate and nitrite in meat reveals its response to nitrosative stress. Front. Microbiol..

[36] Smyth G.K. (2004). Linear models and empirical bayes methods for assessing differential expression in microarray experiments. Stat. Appl. Genet. Mol. Biol..

[37] Livak K., Schmittgen T. (2001). Analysis of relative gene expression data using real-time quantitative PCR and the 2-ΔΔCT method. Methods.

[38] Schlafer S., Meyer R.L. (2017). Confocal microscopy imaging of the biofilm matrix. J. Microbiol. Methods.

[39] Vilain S., Pretorius J.M., Theron J., Brözel V.S. (2009). DNA as an adhesin: *Bacillus cereus* requires extracellular DNA to form biofilms. Appl. Environ. Microbiol..

[40] Zetzmann M., Okshevsky M., Endres J., Sedlag A., Caccia N., Auchter M., Waidmann M.S., Desvaux M., Meyer R.L., Riedel C.U. (2015). DNase-Sensitive and -Resistant Modes of Biofilm Formation by *Listeria monocytogenes*. Front. Microbiol..

[41] Pakkulnan R., Anutrakunchai C., Kanthawong S., Taweechaisupapong S., Chareonsudjai P., Chareonsudjai S. (2019). Extracellular DNA facilitates bacterial adhesion during *Burkholderia pseudomallei* biofilm formation. PLoS ONE.

[42] Regina V.R., Lokanathan A.R., Modrzyński J.J., Sutherland D.S., Meyer R.L. (2014). Surface physicochemistry and ionic strength affects eDNA’s role in bacterial adhesion to abiotic surfaces. PLoS ONE.

[43] Deghorain M., Bobay L.M., Smeesters P.R., Bousbata S., Vermeersch M., Perez-Morga D., Dreze P.A., Rocha E.P., Touchon M., Van Melderen L. (2012). Characterization of Novel Phages Isolated in Coagulase-Negative Staphylococci Reveals Evolutionary Relationships with *Staphylococcus aureus* phages. J. Bacteriol..

[44] Deghorain M., Van Melderen L. (2012). The Staphylococci phages family: An overview. Viruses.

[45] Levine M., Truesdell S., Ramakrishnan T., Bronson M.J. (1975). Dual control of lysogeny by bacteriophage P22: An antirepressor locus and its controlling elements. J. Mol. Biol..

[46] Carrolo M., Frias M.J., Pinto F.R., Melo-Cristino J., Ramirez M. (2010). Prophage Spontaneous Activation Promotes DNA Release Enhancing Biofilm Formation in *Streptococcus pneumoniae*. PLoS ONE.

[47] Gödeke J., Paul K., Lassak J., Thormann K.M. (2011). Phage-induced lysis enhances biofilm formation in *Shewanella oneidensis* MR-1. ISME J..

[48] Binnenkade L., Teichmann L., Thormann K.M. (2014). Iron triggers λSo prophage induction and release of extracellular DNA in *Shewanella oneidensis* MR-1biofilms. Appl. Environ. Microbiol..

[49] Thomas V.C., Sadykov M.R., Chaudhari S.S., Jones J., Endres J.L., Widhelm T.J., Ahn J.S., Jawa R.S., Zimmerman M.C., Bayles K.W. (2014). A central role for carbon-overflow pathways in the modulation of bacterial cell death. PLoS Pathog..

[50] Otto M. (2008). Staphylococcal biofilms. Curr. Top. Microbiol. Immunol..

[51] Dubrac S., Boneca I.G., Poupel O., Msadek T. (2007). New insights into the WalK/WalR (YycG/YycF) essential signal transduction pathway reveal a major role in controlling cell wall metabolism and biofilm formation in *Staphylococcus aureus*. J. Bacteriol..

[52] Gajdiss M., Monk I.R., Bertsche U., Kienemund J., Funk T., Dietrich A., Hort M., Sib E., Stinear T.P., Bierbaum G. (2020). YycH and YycI Regulate Expression of *Staphylococcus aureus* Autolysins by Activation of WalRK Phosphorylation. Microorganisms.

[53] McKenzie G.J., Magner D.B., Lee P.L., Rosenberg S.M. (2003). The *dinB* Operon and Spontaneous Mutation in *Escherichia coli*. J. Bacteriol..

[54] Ayora S., Carrasco B., Cardenas P.P., Cesar C.E., Canas C., Yadav T., Marchisone C., Alonso J.C. (2011). Double-strand break repair in bacteria: A view from *Bacillus subtilis*. FEMS Microbiol. Rev..

[55] Yao Y., Daniel E., Sturdevant D.E., Otto M. (2005). Genome wide analysis of gene expression in *Staphylococcus epidermidis* biofilms: Insights into the pathophysiology of *S. epidermidis* biofilms and the role of phenol-soluble modulins in formation of biofilms. J. Infect. Dis..

[56] Hollis T., Lau A., Ellenberger T. (2000). Structural studies of human alkyladenine glycosylase and *E. coli* 3-methyladenine glycosylase. Mutat. Res. DNA Repair.

[57] Iyer L.M., Zhang D., Rogozin I.B., Aravind L. (2011). Evolution of the deaminase fold and multiple origins of eukaryotic editing and mutagenic nucleic acid deaminases from bacterial toxin systems. Nucleic Acids Res..

[58] Domingues S., Moreira R.N., Andrade J.M., Dos Santos R.F., Bárria C., Viegas S.C., Arraiano C.M. (2015). The role of RNase R in trans-translation and ribosomal quality control. Biochimie.

[59] Schuman W., Hecker M., Msadek T. (2002). Regulation and function of heat-inducible genes in *Bacillus subtilis*. Bacillus subtilis and Its Closest Relatives.

[60] Wang P., Dalbey R.E. (2011). Inserting membrane proteins: The YidC/Oxa1/Alb3 machinery in bacteria, mitochondria, and chloroplasts. Biochim. et Biophys. Acta (BBA)—Biomembr..

[61] Switzer R.L., Zalkin H., Saxild H.H. (2002). Purine, Pyrimidine, and Pyridine Nucleotide Metabolism. Bacillus subtilis and Its Closest Relatives.

[62] Leroy S., Vermassen A., Ras G., Talon R. (2017). Insight into the genome of *Staphylococcus xylosus*, a ubiquitous species well adapted to meat products. Microorganisms.

[63] Beenken K.E., Dunman P.M., McAleese F., Macapagal D., Murphy E., Projan S.J., Blevins J.S., Smeltzer M.S. (2004). Global gene expression in *Staphylococcus aureus* biofilms. J. Bacteriol..

[64] Vimr E.R., Kalivoda K.A., Deszo E.L., Steenbergen S.M. (2004). Diversity of Microbial Sialic Acid Metabolism. Microbiol. Mol. Biol. Rev..

[65] Borisov V.B., Gennis R.B., Hemp J., Verkhovsky M.I. (2011). The cytochrome bd respiratory oxygen reductases. Biochim. Biophys. Acta.

[66] Belitsky B.R. (2002). Biosynthesis of amino acids of the glutamate and aspartate families, alanine, and polyamines. Bacillus subtilis and Its Closest Relatives.

[67] Billot-Klein D., Gutmann L., Bryant D., Bell D., van Heijenoort J., Grewal J., Shlaes D.M. (1996). Peptidoglycan synthesis and structure in *Staphylococcus haemolyticus* expressing increasing levels of resistance to glycopeptide antibiotics. J. Bacteriol..

[68] Schleimer N., Kaspar U., Drescher M., Seggewiß J., von Eiff C., Proctor R.A., Peters G., Kriegeskorte A., Becker K. (2018). The Energy-Coupling Factor Transporter Module EcfAA’T, a Novel Candidate for the Genetic Basis of Fatty Acid-Auxotrophic Small-Colony Variants of *Staphylococcus aureus*. Front. Microbiol..

[69] Fiegler H., Brückner R. (1997). Identification of the serine acetyltransferase gene of *Staphylococcus xylosus*. FEMS Microbiol. Lett..

[70] Kelliher J.L., Radin J.N., Kehl-Fie T.E. (2018). PhoPR contributes to *Staphylococcus aureus* growth during phosphate starvation and pathogenesis in an environment-specific manner. Infect. Immun..

[71] Vermassen A., Talon R., Leroy S. (2016). Ferritin, an iron source in meat for *Staphylococcus xylosus*?. Int. J. Food Microbiol..

[72] Haikarainen T., Papageorgiou A.C. (2010). 2010. Dps-like proteins: Structural and functional insights into a versatile protein family. Cell. Mol. Life Sci..

[73] Ma Z., Jacobsen F.E., Giedroc D.P. (2009). Chemistry Controls Bacterial Metal Homeostasis. Chem. Rev..

[74] Rosenstein R., Futter-Bryniok D., Götz F. (1999). The choline-converting pathway in *Staphylococcus xylosus* C2a: Genetic and physiological characterization. J. Bacteriol..

[75] Resch A., Rosenstein R., Nerz C., Götz F. (2005). Differential gene expression profiling of *Staphylococcus aureus* cultivated under biofilm and planktonic conditions. Appl. Environ. Microbiol..

[76] Beloin C., Ghigo J.M. (2005). Finding gene-expression patterns in bacterial biofilms. Trends Microbiol..

