# Supplementary material for: Investigating Extracellular DNA Release in Staphylococcus xylosus Biofilm In Vitro
Source: Microorganisms. 2021 Oct 21;9(11):2192. doi: 10.3390/microorganisms9112192 (PMC8617998; doi:10.3390/microorganisms9112192)
Supplement: Supplementary file 1 [file microorganisms-09-02192-s001.zip › microorganisms-1392259 -supplementary Table S1.pdf]

Table S1: Targeted genes of *Staphylococcus xylosus* for the validation of microarray data by qPCR: expression at 24 h and 48 h in biofilm compared with 9 h.

Gene Id	Gene name	Sequences of primers pairs for qPCR (5'-3')		Mean ratio of expression			
				Microarray		qPCR	
		Forward	Reverse	24 h	48 h	24 h	48 h
SXYL_01276	*grpE*	CAGGAGGACGATTCACAAAATATC	TCTACGCTTATAGTTTTCAAATTCAGC	3.9	4.8	5.6	4.7
SXYL_01277	*dnaK*	TTAGGTACAACAAATTCATGTATTGCA	GATAGATTGGATTGTATTAGGGTTAGTGA	3.4	3.7	2.7	3.6
SXYL_02088	*uvrA*	AGCGTAGATATGTTGAATCATTAAGTG	GGTTGCAACAGTAGATCTTGGA	3.4	2.6	4.8	3.1
SXYL_02089	*uvrB*	CGTTAGAGGCGATGTAGTGG	ACGTGTTACGAAGTGTGAGG	5.2	4.4	5.3	3.4
SXYL_01796	*uvrC*	CCAAGGTTAATTGTTACACGTACAG	AACCGGATAAACACATGGTCC	3.7	2.1	3.2	2.1
SXYL_01727		AAGCTGGACACACTGTTGATTTA	TTCAACGACTATGTCATATTTCTGC	6.7	7.7	8.2	11.4
SXYL_02505	*katA*	CGTCATCTTCACGAAAGTCATATTC	CGCTAGTACACATTATTATCCAAATAG	4.2	5.7	4.3	4.3
SXYL_00366	*cidB*	CTGCGGTAGGCATAGAAGTATC	ATTTCCATAAGTCAAACCTTTCGC	2.1	11.0	1.6	8.1
SXYL_02535	*ahpF*	AACGCACACCAAGTTTCTCA	TTAATGCTTGAACAACGTCAGG	4.5	4.4	3.6	2.7
SXYL_02534	*ahpC*	CCATTCACAGCTAACGCTTATG	ACCTTGTAAATCTTCTAATTCAGTTGG	3.9	4.1	3.3	2.3
SXYL_01054		GCTCGAGGAATTGGAGGATATC	AGAGAATTTAGGGATTTGTCCAGTT	10.7	20.2	19.9	18.1
SXYL_01275	*hrcA*	GTTGTATTCTCATCAGGTCATGTTG	GATTTCACAAAAGCATTCAAGTCATT	3.8	4.6	2.3	3.6
SXYL_00784	*czrA*	GGAAGATTCATTTAACGAACAAACG	TTGGTGAGAAACGTTAGATTGACT	13.8	13.0	23.3	55.5
SXYL_00837		AACAGTAATCGCATCAACTTTAGC	CCTTCTTGAACTGGTGCTTCA	8.7	13.6	51.1	29.3
SXYL_00935	*putP2*	GCGAGTTGGCAGACTTATATTATG	GACCAATACTTCTGCCTCCAA	0.4	0.4	0.4	0.6
SXYL_00326	*copZ*	ATGGCAACTGAAACAATTAAAGTAGA	TTATTTTACGTCATAACCTTGATCCTCTA	8.5	10.2	12.8	13.4
SXYL_01098	*ribE*	TGTTTACAGGAATTGTTGAAGAAATAGG	CTCTTTCTAAATTCACTTCAGCAGAT	3.2	4.4	3.3	5.5
SXYL_01318	*gcvPA*	GAGCAAGATAAGCAGGAAATGTTAG	TGAACGAGAAATCATTGCATCTACTA	3.1	2.4	7.6	2.7
SXYL_01474	*alr2*	ACGCTGAAAATGTAAAACAAGACA	TGAAGTCGTGCTAAATGTATTAATGC	11.9	7.6	18.3	7.4
SXYL_01548	*guaC*	TGAATGAAGAATTAGCTGAATGGTTC	TTTAACGCCTACAGAAATTGATGC	2.5	3.0	2.3	2.9
SXYL_01961	*argG*	TGGTTTAGATACAAGTGTCGCA	TGCATATGACACGAAATCTTCAC	0.2	0.2	0.1	0.1
SXYL_01974	*mnhE1*	CGTCACAGGCAGTTATACATTTAATAA	CGGCTCATTATCAATCTTTGGTTT	0.4	0.5	0.4	0.4
SXYL_02548		GCATTAGAATTTGCCGCAATG	AGCAGCATACTCACCATTCG	7.2	7.5	17.1	12.0
SXYL_00005	*gyrB*	GAAGATTATGGTGCTGGACAGATA	ACGCCCATTATCCGTTACTTTAAT	2.2	2.1	1.5	1.3
SXYL_00555	*scrA*	GAAGATAATGTTGAAGCAATGACACA	TGAAAACGTTCCTTTTACAACATCC	0.2	0.3	0.2	0.3
SXYL_00370		ATGCCAAAAGTATCCTTAGTACCA	GCACGTTCATCTTCAATCGTC	0.3	0.3	0.3	0.2
SXYL_00548		GAAGGTGATCCAACTGAGTTGT	ATGCGACCATCTTCATCTTGTT	7.6	7.3	10.9	9.1
SXYL_00652		GCAATCGTAGGATTAGATCACGG	TACATACCAAGTACATTGTCCTGC	6.0	11.0	18.7	16.0
SXYL_00365	*cidA*	GGCAATGTAATTCAGAGCGTATT	AGAAACGACATCCATAACACCAA	3.4	16.1	3.1	15.2
